# The Safety and Efficacy of a Novel Saw Palmetto ( *Serenoa repens* ) Extract for Promoting Hair Growth in Adults With Self‐Perceived Thinning Hair: 180‐Day Results

**DOI:** 10.1111/jocd.70717

**Published:** 2026-02-06

**Authors:** Glynis Ablon

**Affiliations:** ^1^ Medical, Surgical, and Cosmetic Dermatology Ablon Skin Institute and Research Center Manhattan Beach California USA

**Keywords:** alopecia, clinical trial, hair shedding, male and female pattern hair loss, saw palmetto, *Serenoa repens*, thinning hair

## Abstract

**Background:**

Hair loss remains a global concern for both men and women.

**Aims:**

This study assessed the efficacy and safety of a proprietary extract of bioactive fatty acids from saw palmetto ( *Serenoa repens* ) for treating self‐perceived thinning hair in healthy adult men and women (SEREVELLE, Valensa International; Eustis, FL).

**Methods:**

This 6‐month, randomized, double‐blind, placebo‐controlled study assessed the beneficial effects of daily active treatment (*n* = 40) vs. placebo (*n* = 20) on several hair growth parameters.

**Results:**

Active treatment of SEREVELLE significantly outperformed placebo on all primary endpoints at Day 180. Mean (SD) change in baseline total terminal hair count was +18.6 (29.6) with active vs. –10.1 (30.5) with placebo (*p* < 0.001; 283% greater improvement). Total vellus hair count increased +6.6 (15.6) vs. –2.1 (15.7) (*p* < 0.05; 414% greater improvement). Total hair density rose +25.1 (27.7) vs. –12.2 (38.8) (*p* < 0.001; 306% greater improvement). Subgroup analyses at Day 180 corroborated these advantages: across populations, between‐group differences in mean change favored active for subgroup of men, with total terminal hair count (*p* = 0.003), total vellus hair count (*p* < 0.001), and total hair density (*p* < 0.001). In menopausal women, active produced significant placebo‐adjusted gains in total terminal hair count (*p* < 0.05) and total hair density (*p* < 0.01), with a nonsignificant numerical advantage for total vellus hair count. There were no treatment‐related adverse events.

**Conclusion:**

The daily use of a proprietary saw palmetto ( *Serenoa repens* ) extract safely and effectively promotes hair growth in men and women with self‐perceived thinning hair.

## 1 Introduction

Hair loss has long been a global concern for both men and women [1]. Unfortunately, drug therapy for hair loss remains limited to oral or topical minoxidil for male pattern hair loss to which oral finasteride may be added, if necessary [2]. Similarly, drug therapy can include oral or topical minoxidil for female pattern hair loss to which oral spironolactone may be added in premenopausal women [3] and oral finasteride may be added in postmenopausal women [2].

Oil extracted from saw palmetto berries ( *Serenoa repens* ) contains 80%–85% fatty acids and some minor components like sterolic alcohols [4]. Unlike finasteride's active‐site inhibition, lipidosterolic extracts of *Serenoa repens* inhibit both type I and II 5‐α‐reductase via non‐competitive (allosteric) mechanisms, an effect largely attributed to free fatty acids in the extract [5]. Thus, *S. repens* promotes follicle health and hair growth by blocking the enzyme that converts testosterone to its active metabolite dihydrotestosterone [6]. The efficacy of topical *S. repens* for treating hair loss, such as male androgenetic alopecia, has previously been demonstrated [7, 8]. but there is considerable variability across the saw palmetto products, underscoring the importance of well‐defined composition, standardized fatty acid profile, and consistent quality. In this context, the test product used in this trial (SEREVELLE, Valensa International; Eustis, FL) represents a characterized proprietary bioactive fatty acid extract from saw palmetto.

A novel, proprietary *Serenoa repens* fatty‐acid extract of SEREVELLE containing ActiLipid4Hair has been developed for promoting hair growth, follicular reactivation, and hair health. The product is available in a concentrated oral form with beneficial effects on overall hair health, hair follicle reactivation, and inhibiting hair thinning by maintaining a longer hair growth phase [7].

A 6‐month, randomized, double‐blind, placebo‐controlled study assessed the efficacy of SEREVELLE to promote hair growth in healthy adult men and women (*N* = 60) with self‐perceived hair thinning. Presented here are the results of a 180‐day assessment, evaluating the long‐term effects of a bioactive fatty‐acid *Serenoa repens* extract on hair growth in adults with self‐perceived hair thinning.

## 2 Methods

### 2.1 Study Population

Eligible participants were healthy men and women, aged 25–65 years, of any Fitzpatrick Skin Type, who reported concerns about hair thinning and expressed interest in improving hair density and appearance. Male subjects qualified if they presented with hair loss patterns I, II, IIA, III, III vertex, or IV on the Investigator‐rated Norwood Classification Scale [9]. Female subjects were required to have thinning patterns corresponding to grades I‐1, I‐2, or I‐3 on the Savin Pictorial Scale [10].

All subjects agreed to maintain their current hairstyle, length, and color; adhere to their normal diet and medication routines; attend all scheduled visits; undergo brief physical examinations and digital scalp imaging; and follow all study procedures for the full duration of participation.

Subjects were excluded if they had undergone hair transplantation or used extensions, experienced a major stressor within 6 months, or had within 3 months begun or altered hormonal therapy, used minoxidil, finasteride, low‐level laser therapy, or any prescription medication known to affect the hair growth cycle, as well as a stressful incident within the last 6 months. Other exclusion criteria included active scalp disease, autoimmune or systemic disorders, immune deficiency, hepatitis, pregnancy or lactation, or participation in another investigational study.

### 2.2 Investigational Product

Subjects were randomized to receive the investigational supplement or placebo. The active intervention consisted of a proprietary bioactive fatty acid extract of *Serenoa repens* (saw palmetto) enriched in four key free fatty acids (ActiLipid4Hair) with potent inhibitory effects on 5α‐reductase‐1 activity [11]. SEREVELLE contains the lipid fraction of saw palmetto fruit. The dried ripe berries are milled using a cryogenic milling process. The bioactive lipids are then extracted using a proprietary DeepExtract supercritical carbon dioxide process without the use of cosolvents. The resulting extract contains fatty acids, 0.20 to 0.50% sterols, and 0.15% to 0.35% long‐chain alcohols. The active compounds are the unbound (free) fatty acids. The molecular weight of fatty acids ranges from 116 to 284 Da, with a weighted mean of 237 Da. The formulation contained 160 mg of the concentrated extract per softgel (SEREVELLE, Valensa International, Eustis, FL) delivering 105 mg of ActiLipid4Hair bioactive fatty acids with a soft gel capsule matrix of bovine gelatin, glycerin, and water. The placebo capsules were identical in appearance and contained palm oil. Subjects were instructed to ingest one capsule daily with water.

### 2.3 Study Design and Endpoints

This randomized, double‐blind, placebo‐controlled trial extended over 180 days. Subjects were assigned after randomization to receive either active product (*n* = 40) or placebo (*n* = 20) and were evaluated at baseline (Day 0), Day 90, and Day 180.

The three primary endpoints included the change from baseline in terminal, vellus, and total hair counts at anterior and posterior target regions (per cm^2^) compared with placebo.

Secondary endpoints assessed the terminal‐to‐vellus ratio, mean hair shaft width, mean number of hairs per follicular unit, follicular units/cm^2^, mean interfollicular distance, and investigator‐rated global scales for hair growth and quality. Additional self‐reported outcomes included the Hair Self‐Assessment Questionnaire, Hair Thinning Quality‐of‐Life Questionnaire, and Hair Product Satisfaction Questionnaire.

### 2.4 Safety

Safety assessments included adverse event monitoring and product tolerability. As saw palmetto extracts have an extensive history of oral use and safety in numerous published reports including randomized trials, this protocol prioritized clinical safety monitoring rather than clinical hematology and chemistry panels. Specifically, subject safety was monitored through medical history and eligibility screening, excluding subjects with clinically relevant health conditions; systematic adverse event (AE) surveillance at each study visit using open‐ended questioning and structured AE documentation; vital signs and general clinical assessments at each visit; and documentation of concomitant medications and discontinuations/withdrawals due to tolerability issues.

### 2.5 Imaging and Assessments

Two‐dimensional standardized images of the scalp were captured at each site visit 1 (Day 0), Visit 2 (Day 90), and Visit 3 (Day 180) using the IntelliStudio System (Canfield Scientific, Parsippany, NJ). For quantitative trichoscopy, two 1‐cm^2^ target areas were selected: one at the frontal hairline near the frontalis region where the frontal and lateral hairlines meet and one posteriorly in the transitional scalp zone. The sites were denoted with a black marker. These locations were chosen to represent active thinning areas adjacent to healthier hair. HairMetrix software (Canfield Scientific, Parsippany, NJ) quantified hair density, diameter, and follicular metrics from these sites.

Global hair growth and quality were rated by the investigator looking at hair brittleness, dryness, texture, shine, scalp coverage, and overall appearance using a 7‐point Likert scale, referencing both standardized photographs and in‐person evaluations. Subjects completed validated self‐assessed questionnaires of perceived changes in hair characteristics and their quality of life related to their perceived thinning hair at baseline, 90 days, and 180 days.

### 2.6 Statistical Analysis

Analyses compared within‐ and between‐group changes from baseline to Day 180, looking separately at the three primary efficacy endpoints which were the change in anterior and posterior hair counts for terminal hair, vellus hair, and total hair counts. Continuous variables were analyzed using paired or unpaired two‐tailed Student's *t*‐tests as appropriate. The primary comparison assessed mean differences in hair count changes between active and placebo groups.

### 2.7 Ethical Conduct

All subjects provided written informed consent, including HIPAA and photography releases, prior to enrollment. The protocol was approved by Allendale IRB (Old Lyme, CT), a commercial institutional review board, and conducted in compliance with FDA 21 CFR Part 50 and Good Clinical Practice (GCP) standards for human research. The study is registered under ClinicalTrials.gov identifier NCT06920758.

## 3 Results

The enrolled male (*n* = 30) and female (*n* = 30) subjects had a mean age of 50.0 years and were mostly White (83%), non‐Hispanic (78%), and evenly distributed across Fitzpatrick Skin Types I through IV (Table 1). The male subjects had male pattern hair loss predominantly of Norwood Classification Scale Type III (50%) and III Vertex (43%), while the female subjects had female pattern hair loss predominantly of Savin Pictoral Scale Grade I‐2 (73%).

**TABLE 1:** Subject demographics and clinical characteristics.

	Active, *n* = 40	Placebo, *n* = 20	ALL, *N* = 60
Mean age, years (SD)	50.6 (9.6)	48.9 (10.9)	50.0 (10.0)
Median age, years (min, max)	55.0 (28, 63)	50.5 (29, 63)	52 (28, 63)
Race, *n* (%)	*n* = 40	*n* = 20	*N* = 60
White	36 (90)	14 (70)	50 (83)
Asian	1 (2.5)	2 (10)	3 (5)
Black	1 (2.5)	1 (5)	2 (3)
American indian/alaskan native		2 (10)	2 (3)
Native hawaiian/pacific islander		1 (5)	1 (2)
White/african american	1 (2.5)		1 (2)
White/african american/native american	1 (2.5)		1 (2)
Ethnicity, *n* (%)	*n* = 40	*n* = 20	*N* = 60
Hispanic/Latino	32 (80)	15 (75)	13 (22)
Non‐hispanic/Latino	8 (20)	5 (25)	47 (7%)
Fitzpatrick skin type, *n* (%)	*n* = 40	*n* = 20	*N* = 60
Type I	6 (15)	5 (25)	11 (18)
Type II	14 (35)	4 (20)	18 (30)
Type III	12 (30)	5 (25)	17 (28)
Type IV	6 (15)	5 (25)	11 (18)
Type V	2 (5)		2 (4)
Type VI		1 (5)	1 (2)
Norwood classification scale (male subjects), *n* (%)	*n* = 20	*n* = 10	*N* = 30
II	1 (5)	1 (10)	2 (7)
III	8 (40)	7 (70)	15 (50)
III Vertex	11 (55)	2 (20)	13 (43)
Savin pictoral scale (female subjects), *n* (%)	*n* = 20	*n* = 10	*N* = 30
1–2	15 (75)	7 (70)	22 (73)
1–3	5 (25)	3 (30)	8 (27)

*Note:* There were no significant between‐group differences.

### 3.1 Primary Endpoints: All Subjects

#### 3.1.1 Terminal Hair Counts

By Day 180, the mean (SD) anterior terminal hair count in the active treatment group increased by 5.5 (23.8), whereas placebo fell below baseline by 180 days −2.5 (22.8) (*p* = NS). The largest mean growth effects were localized in posterior terminal hair count, which increased significantly by 13.0 (22.4) at Day 180 for the active group (*p* < 0.001), while the mean posterior terminal hair counts for the placebo group decreased by −7.6 (18.4). At Day 180, the mean change in total terminal hair count was 18.6 (29.6) for the active group and −10.1 (30.5) for the placebo group (*p* < 0.001) (Table 2) corresponding to 283% greater improvement in the active treatment group compared to placebo.

**TABLE 2:** Primary endpoint, hair counts by treatment group.

	Active treatment, *n* = 40		Placebo, *n* = 20	
	Day 0	Day 180	Day 0	Day 180
*Anterior terminal hair counts*				
Mean (SD)	132.9 (29.2)	138.5 (30.1)	131.8 (30.7)	129.3 (29.1)
Median (min, max)	134.5 (60, 202)	136 (84, 214)	137.5 (85, 199)	136 (74, 168)
Change (SD)		5.5 (23.8)		−2.5 (22.8)
*Posterior terminal hair counts*				
Mean (SD)	167.1 (32.9)	180.1 (40.2)^a^, ^b^	175.2 (34.5)	167.6 (31.5)
Median (min, max)	168 (100, 233)	180 (80, 260)	175 (123, 246)	165.5 (118, 220)
Change (SD)		13.0 (22.4)		−7.6 (18.4)
*Total terminal hair counts*				
Mean (SD)	300.0 (46.1)	318.6 (48.5)^a^, ^b^	306.9 (59.6)	296.8 (55.8)
Median (min, max)	302.5 (185, 426)	305.0 (244, 459)	307.5 (212, 420)	300.0 (193, 388)
Change (SD)		18.6 (29.6)		–10.1 (30.5)
*Anterior vellus hair counts*				
Mean (SD)	10.8 (9.5)	14.4 (12.1)^c^	14.9 (10.9)	11.1 (9.4)
Median (min, max)	8 (0, 37)	10 (0, 51)	14 (0, 35)	9 (0, 43)
Change (SD)		3.6 (11.3)		−3.9 (13.1)
*Posterior vellus hair counts*				
Mean (SD)	4.8 (6.6)	7.8 (7.3)^d^	3.5 (3.1)	5.3 (5.8)
Median (min, max)	1 (0, 29)	6 (0, 26)	3 (0, 11)	3 (0, 17)
Change (SD)		3.0 (7.7)		1.7 (6.8)
*Total vellus hair counts*				
Mean (SD)	15.5 (13.8)	22.1 (15.0)^c^	18.4 (11.7)	16.3 (13.1)
Median (min, max)	11.5 (0, 55)	20.0 (0, 57)	18.0 (0, 41)	14.5 (0, 57)
Change (SD)		6.6 (15.6)		–2.1 (15.7)
*Anterior total hair density*				
Mean (SD)	143.7 (28.0)	152.9 (32.6)^c^	146.7 (33.3)	140.3 (27.7)
Median (min, max)	146.5 (66, 206)	154 (87, 220)	142 (98, 222)	149.5 (83, 174)
Change (SD)		9.2 (21.6)		−6.3 (28.4)
*Posterior total hair counts*				
Mean (SD)	171.9 (35.4)	187.9 (39.0)^a^, ^b^	178.7 (33.3)	172.8 (32.3)
Median (min, max)	171 (100, 246)	194 (106, 269)	178 (130, 249)	174 (125, 227)
Change (SD)		16.0 (21.9)		−5.8 (20.5)
*Total hair counts*				
Mean (SD)	315.6 (48.9)	340.7 (51.0)^a^, ^b^	325.3 (60.5)	313.1 (5.7)
Median (min, max)	318.0 (191, 452)	344.0 (255, 471)	317.50 (239, 436)	313.5 (208, 394)
Change (SD)		25.1 (27.7)		−12.2 (38.8)

^a^ *p* < 0.05 versus baseline.

^b^ 2‐tailed Student's *t*‐tests for correlated samples *p* < 0.001.

^c^ 2‐tailed Student's *t*‐tests for correlated samples *p* < 0.05.

^d^ *p* < 0.01 versus baseline.

#### 3.1.2 Vellus Hair Counts

The mean (SD) anterior vellus hair count from Day 0 increased by 3.6 (11.3) at Day 180 for subjects in the active treatment group and decreased by 3.9 (13.1) for subjects in the placebo group with a significant between‐group difference (7.46) in mean change in vellus hair counts at Day 180 (*p* < 0.05). The mean posterior vellus hair count increased by 3.0 (7.7) for the active treatment group and by 1.8 (6.8) for the placebo group at Day 180. While the difference was not statistically significant between groups, there was a significant increase in hair count from baseline for active treatment groups. At Day 180, the mean change in total vellus hair count was 6.6 (15.6) for the active group and –2.1 (15.7) for the placebo group, reflecting a 414% higher increase in vellus hair count in the active treatment group compared to placebo (*p* < 0.05) (Table 2).

#### 3.1.3 Total Hair Density

Results of the sum of terminal and vellus hair counts were expressed as Total Hair Density. There was a significant increase in mean total anterior hair density for the active treatment group at Day 180 of 9.2 (21.6) but a decrease for the placebo group of −6.3 (28.4) with a significant between‐group difference for mean change in total hair density (*p* < 0.05). There was a significant increase of 16.0 (21.9) in total posterior hair density from Day 0 to Day 180 for the active group compared with a mean decrease in hair count of −5.9 (20.5) in the placebo group, and the difference between groups was significant (*p* < 0.001). The mean increase in total hair density was 25.1 (27.7) for the active treatment group vs. a decrease of −12.2 (38.8) for the placebo group, corresponding to 306% improved growth in total hair density in the active treatment group compared to placebo, with the between‐group difference also being statistically significant (*p* < 0.001) (Table 2).

**FIGURE 1:**
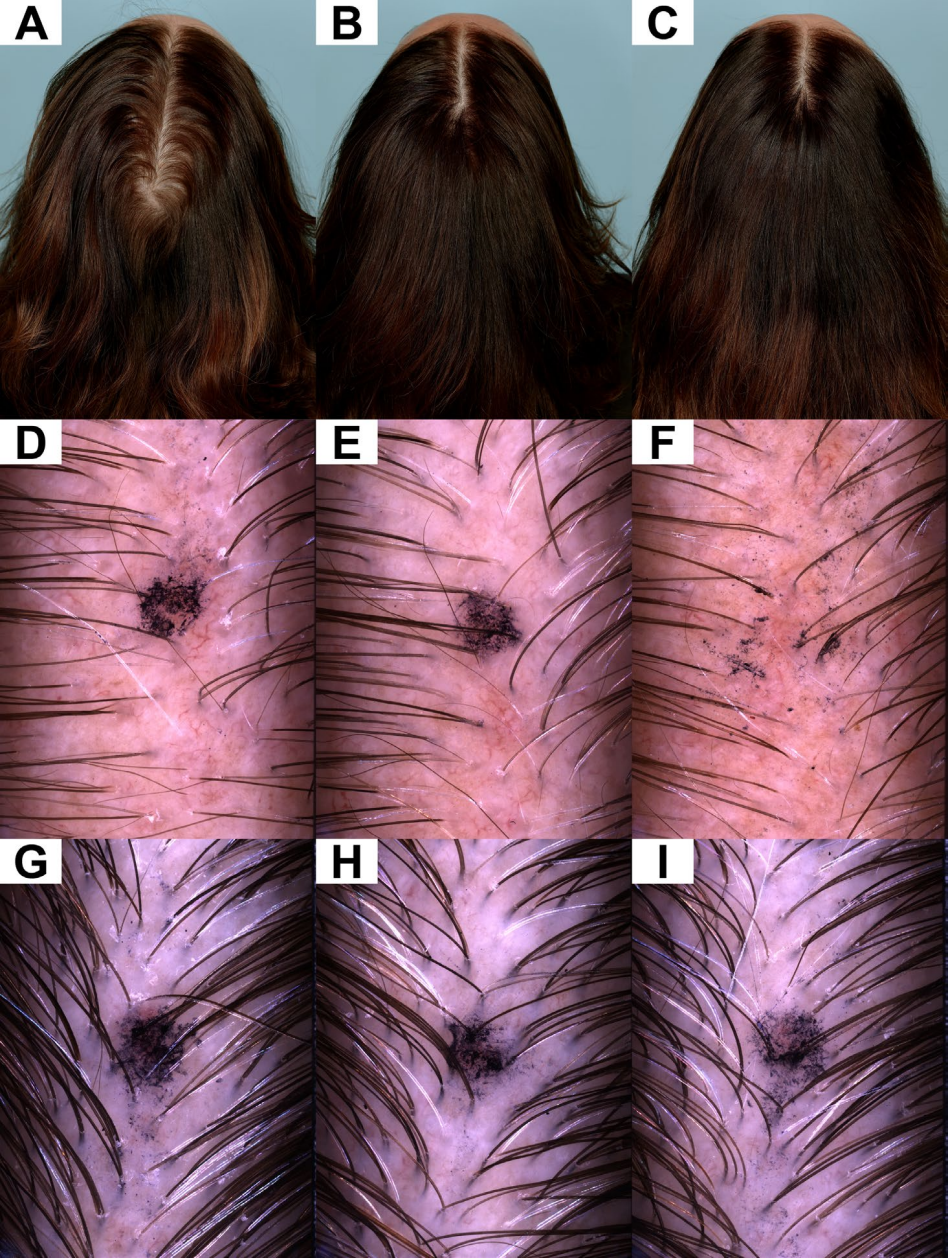
The change in hair appearance of this female subject is apparent from Baseline (A) to Day 90 (B) and Day 180 (C). At Baseline, the subject displayed a right temporal terminal:vellus ratio of 14.0:1, a mean 1.3 hairs per follicular unit, 107.0 follicular units per cm^2^, mean inter‐follicular distance of 1.17 mm, mean hair width of 59.5 μm, total hair count of 154/cm^2^, and total (sum) hair width of 9179 μm/cm^2^ (D). The subject displayed a center vertex mean 1.6 hairs per follicular unit, 110.0 follicular units per cm^2^, mean inter‐follicular distance of 1.09 mm, mean hair width of 66.2 μm, total hair count of 180/cm^2^, and total hair width of 11 914 μm/cm^2^ (G). At Day 90, the subject displayed a right temporal terminal:vellus ratio of 8.0:1, a mean 1.4 hairs per follicular unit, 105.0 follicular units per cm^2^, mean inter‐follicular distance of 1.17 mm, mean hair width of 57.9 μm, total hair count of 165/cm^2^, and total (sum) hair width of 9590 μm/cm^2^ (E). The subject displayed a center vertex temporal mean 1.8 hairs per follicular unit, 111.0 follicular units per cm^2^, mean inter‐follicular distance of 1.17 mm, mean hair width of 65.7 μm, total hair count of 196/cm^2^, and total hair width of 12 891 μm/cm^2^ (H). By Day 180, the subject had achieved a right temporal terminal:vellus ratio of 12.5:1, a mean 1.3 hairs per follicular unit, 108.0 follicular units per cm^2^, mean inter‐follicular distance of 1.24 mm, mean hair width of 54.8 μm, total hair count of 153/cm^2^, and total (sum) hair width of 8432 μm/cm^2^ (F). The subject displayed a center vertex temporal mean 1.9 hairs per follicular unit, 122.0 follicular units per cm^2^, mean inter‐follicular distance of 1.10 mm, mean hair width of 65.6 μm, total hair count of 231/cm^2^, and total hair width of 15 144 μm/cm^2^ (I).

**FIGURE 2:**
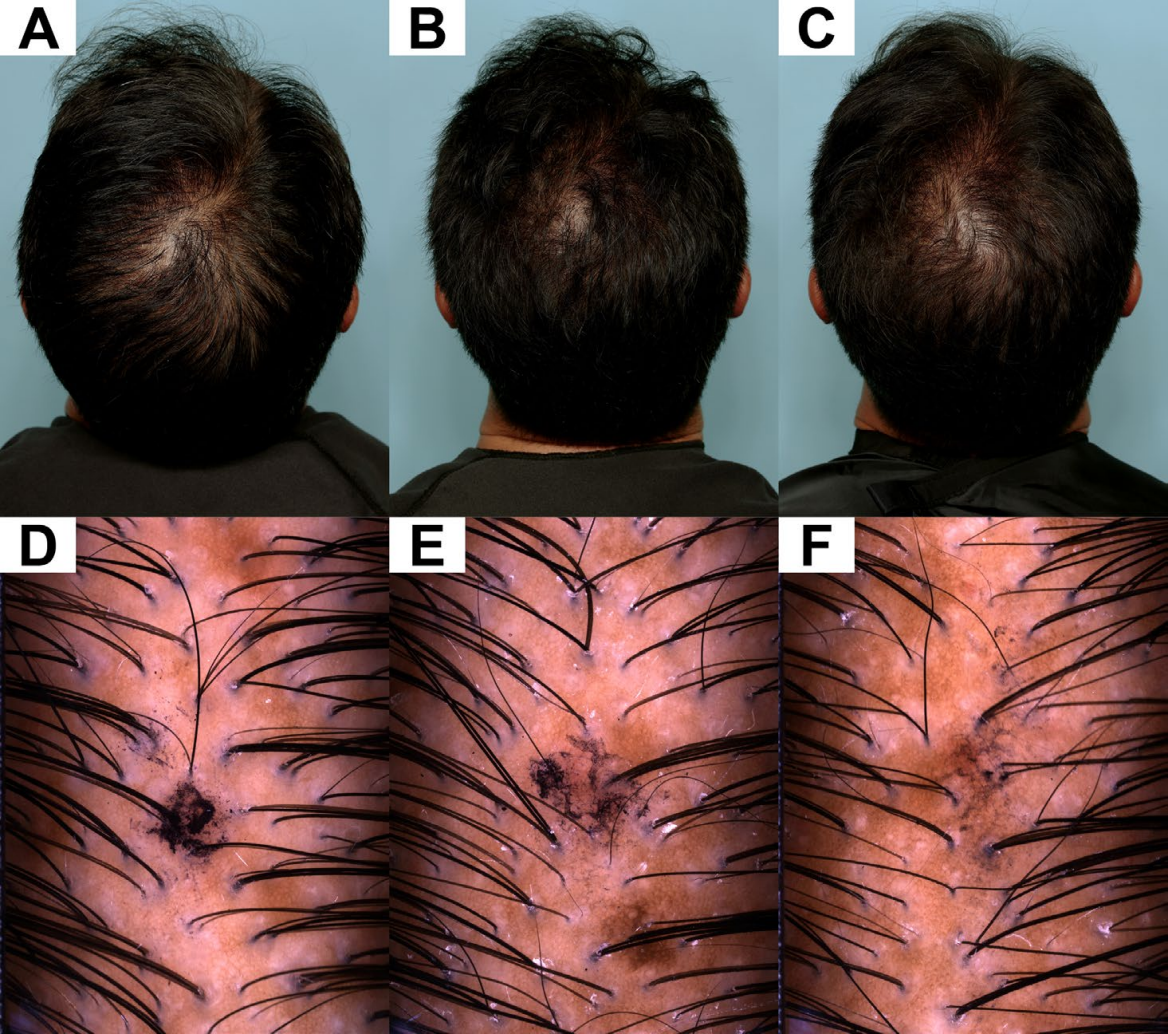
The change in hair appearance of this male subject is apparent from Baseline (A) to Day 90 (B) and Day 180 (C). At Baseline, the subject displayed a right frontal ratio of 23.5:1, a mean 1.4 hairs per follicular unit, 96.0 follicular units per cm^2^, mean inter‐follicular distance of 1.2 mm, mean hair width of 56.2 μm, total hair count of 140/cm^2^, and total hair width of 7846 μm/cm^2^. The center vertex area had a mean 1.5 hairs per follicular unit, 116.0 follicular units per cm^2^, mean inter‐follicular distance of 1.19 mm, mean hair width of 60.2 μm, total hair count of 177/cm^2^, and total hair width of 10 634 μm/cm^2^ (D). At Day 90, the subject displayed a right frontal temporal terminal:vellus ratio of 13.3:1, a mean 1.4 hairs per follicular unit, 101.0 follicular units per cm^2^, mean inter‐follicular distance of 1.2 mm, mean hair width of 56.5 μm, total hair count of 148/cm^2^, and total hair width of 8351 μm/cm^2^. The center vertex area had a right frontal temporal terminal:vellus ratio of 12.2:1, a mean 1.4 hairs per follicular unit, 118.0 follicular units per cm^2^, mean inter‐follicular distance of 1.12 mm, mean hair width of 58.6 μm, total hair count of 177/cm^2^, and total hair width of 10 384 μm/cm^2^ (**E**). By day 180, the subject had achieved a right frontal temporal terminal:vellus ratio of 10.4, a mean 1.2 hairs per follicular unit, 119.0 follicular units per cm^2^, mean inter‐follicular distance of 1.2 mm, mean hair width of 54.1 μm, total hair count of 162/cm^2^, and total hair width of 8794 μm/cm^2^. The subject displayed a center vertex temporal terminal:vellus ratio of 10.3:1, mean 1.5 hairs per follicular unit, 116.0 follicular units per cm^2^, mean inter‐follicular distance of 1.21 mm, mean hair width of 53.2 μm, total hair count of 194/cm^2^, and total hair width of 10 313 μm/cm^2^ (**F**).

Overall, the primary endpoint analysis revealed statistically significant and clinically meaningful changes in posterior terminal, anterior vellus, anterior and posterior hair density, as well as for total terminal, total vellus, and total hair density at study Day 180 for the active treatment group versus placebo, strongly supporting the positive long‐term treatment effect on hair growth in men and women. Representative pre‐ and posttreatment images of a female and male subject are provided as Figure 1 and Figure 2, respectively.

### 3.2 Primary Endpoints: Male Subjects

Changes from baseline favored active treatment across all scalp hair measures. Compared with placebo, the active treatment group showed statistically significant increases in total terminal hair count (between‐group difference in mean change; *p* < 0.005), total vellus hair count (*p* < 0.001), and total hair density (*p* < 0.001). These effects corresponded to a 154% (2.5‐fold) greater improvement in total terminal hair count, a 378% (4.7‐fold) greater improvement in total vellus hair count, and a 192% (3‐fold) greater improvement in total hair density with active treatment vs. placebo. All *p*‐values refer to between‐group differences in the mean change from baseline to Day 180 Table 3.

**TABLE 3:** Primary endpoint, hair counts for male treatment subgroups.

	Active treatment, *n* = 40		Placebo, *n* = 20	
	Day 0	Day 180	Day 0	Day 180
*Anterior terminal hair counts*				
Mean (SD)	144.4 (25.7)	150.5 (30.2)	139.3 (35.0)	131.4 (32.6)
Median (min, max)	147.0 (91, 202)	142.0 (107, 214)	137.5 (97, 199)	138 (74, 168)
*Posterior terminal hair counts*				
Mean (SD)	151.8 (33.17)	159.3 (37.8)^a^	181.9 (38.7)	164.8 (38.0)
Median (min, max)	148.5 (100, 233)	155.5 (80, 245)	184.5 (123, 246)	165.5 (118, 220)
*Total terminal hair counts*				
Mean (SD)	296.2 (48.4)	309.9 (52.5)^b^	321.2 (66.7)	296.2 (65.1)
Median (min, max)	292.0 (222, 426)	289.5 (250, 459)	324.5 (222, 420)	292.5 (193, 388)
*Anterior vellus hair counts*				
Mean (SD)	7.1 (7.7)	15.1 (12.4)	11.2 (11.4)	7.1 (5.8)
Median (min, max)	5.0 (0, 30)	11.0 (0, 51)	6.0 (0, 31)	6.0 (0, 17)
*Posterior vellus hair counts*				
Mean (SD)	4.2 (5.0)	10.4 (7.8)^a^, ^d^	4.7 (3.0)	3.7 (4.1)
Median (min, max)	2.5 (0, 14)	9.0 (0, 26)	4.0 (0, 11)	3.0 (0, 14)
*Total vellus hair counts*				
Mean (SD)	11.3 (11.2)	25.5 (15.4)^e^, ^f^	15.9 (12.9)	10.8 (8.0)
Median (min, max)	9.5 (0, 44)	21.5 (0, 57)	11.5 (0, 38)	10.5 (0, 22)
*Anterior total hair counts*				
Mean (SD)	151.5 (24.4)	165.7 (30.3)^c^	150.5 (40.6)	138.5 (31.2)
Median (min, max)	149.5 (115, 206)	160.0 (122, 220)	150.5 (105, 222)	138.5 (83, 174)
*Posterior total hair counts*				
Mean (SD)	156.0 (34.3)	169.7 (37.5)^f^	186.6 (37.4)	168.5 (36.5)
Median (min, max)	157.5 (100, 246)	168.5 (106, 251)	188.5 (134, 249)	167.0 (125, 223)
*Total hair counts*				
Mean (SD)	307.6 (50.5)	335.4 (56.0)^f^	337.1 (69.8)	307.0 (63.1)
Median (min, max)	308.0 (233, 452)	328.5 (267, 471)	335.0 (239, 436)	302.0 (208, 394)

^a^ 2‐tailed Student's *t*‐tests for correlated samples *p* < 0.01.

^b^ 2‐tailed Student's *t*‐tests for correlated samples *p* < 0.005.

^c^ 2‐tailed Student's *t*‐tests for correlated samples *p* < 0.05.

^d^ *p* < 0.01 versus baseline.

^e^ *p* < 0.001 versus baseline.

^f^ 2‐tailed Student's *t*‐tests for correlated samples *p* < 0.001.

### 3.3 Primary Endpoints: Menopausal Female Subjects

In this subgroup, outcomes were analyzed as the between‐group difference in mean change from baseline (active vs. placebo). Relative to placebo, the active treatment group produced a significant increase in total terminal hair count (*p* < 0.05) and total hair density (*p* < 0.01), corresponding to a 312% (4‐fold) greater improvement in total terminal hair count and a 230% (3‐fold) greater improvement in total hair density over the 180‐day period. For vellus hair count, the placebo‐adjusted change was 117% (2‐fold) higher in the active treatment group and numerically favored active treatment but did not reach statistical significance by the end of the trial Table 4.

**TABLE 4:** Primary endpoint, hair counts for menopausal female treatment subgroups.

	Active treatment, *n* = 40		Placebo, *n* = 20	
	Day 0	Day 180	Day 0	Day 180
*Anterior terminal hair counts*				
Mean (SD)	128.6 (23.7)	130.0 (22.9)	120.6 (26.2)	115.8 (30.4)
Median (min, max)	128.0 (82, 169)	130.0 (87, 168)	126.0 (88, 147)	102.0 (83, 156)
*Posterior terminal hair counts*				
Mean (SD)	185.8 (23.9)	204.8 (31.5)^a^, ^b^	169.6 (43.5)	164.8 (33.3)
Median (min, max)	194.0 (142, 220)	210.0 (142, 260)	159.0 (124, 236)	165.0 (123, 210)
*Total terminal hair counts*				
Mean (SD)	314.4 (34.2)	334.8 (38.9)^c^	290.2 (68.1)	280.6 (±59.6)
Median (min, max)	321.0 (252, 375)	342.0 (272, 380)	285.0 (212, 383)	267.0 (222, 349)
*Anterior vellus hair counts*				
Mean (SD)	13.6 (9.8)	14.4 (11.9)	24.2 (10.0)	13.2 (2.5)
Median (min, max)	12.0 (0, 37)	11.0 (0, 43)	20.0 (15, 35)	14.0 (9, 16)
*Posterior vellus hair counts*				
Mean (SD)	5.4 (8.8)	5.8 (6.2)	2.8 (2.7)	6.8 (6.4)
Median (min, max)	0.0 (0, 29)	6.0 (0, 23)	3.0 (0, 6.0)	6.0 (0, 17)
*Total vellus hair counts*				
Mean (SD)	19.0 (15.6)	20.2 (14.5)	27.0 (10.2)	20.0 (4.8)
Median (min, max)	13.0 (0, 55)	15.0 (0, 52)	20.0 (19, 41)	22.0 (14, 26)
*Anterior total hair counts*				
Mean (SD)	142.2 (23.1)	144.5 (24.6)^c^	144.8 (27.8)	129.0 (29.4)
Median (min, max)	145.0 (96, 178)	153.0 (95, 179)	141.0 (115, 182)	115.0 (97, 170)
*Posterior total hair counts*				
Mean (SD)	191.3 (28.9)	210.6 (32.4)^c^, ^d^	172.4 (41.2)	171.6 (38.4)
Median (min, max)	194.0 (142, 238)	210.0 (145, 269)	164.0 (130, 236)	168.0 (129, 227)
*Total hair counts*				
Mean (SD)	333.5 (34.8)	355.1 (36.3)^e^	317.2 (68.1)	300.6 (63.0)
Median (min, max)	334.0 (266, 381)	357.0 (188, 407)	305.0 (253, 418)	283.0 (242, 375)

^a^ 2‐tailed Student's *t*‐tests for correlated samples *p* < 0.01.

^b^ *p* < 0.05 versus baseline.

^c^ 2‐tailed Student's *t*‐tests for correlated samples *p* < 0.05.

^d^ *p* < 0.01 versus baseline.

^e^ 2‐tailed Student's *t*‐tests for correlated samples *p* < 0.001.

### 3.4 Secondary Endpoints, All Subjects

Unless otherwise noted, there were no between‐group differences for any secondary endpoint on Day 0. For the total sum of hair width, the mean change from baseline increased by 1759.8 μm in the active treatment group and decreased by 538.4 μm in the placebo group by Day 180, showing a significant between‐group difference (*p* < 0.01). This corresponded to approximately a 436% (5‐fold) greater improvement in total hair width with active treatment vs. placebo. This effect was driven by the posterior region, where the mean change rose by 849.7 μm with active vs. a decrease of 397.3 μm with placebo, yielding a significant between‐group difference (*p* < 0.05). In the anterior region, the mean change increased by 435.4 μm with active treatment and decreased by 141.2 μm with placebo Table 5.

**TABLE 5:** Secondary endpoint, sum of hair width and number of follicular units/cm^2^ for each treatment group.

Sum of hair width	Active treatment, *n* = 40		Placebo, *n* = 20	
Anterior sum of hair width	**Day 0**	**Day 180**	**Day 0**	**Day 180**
Mean (SD)	8972.6 (1898)	9408 (1944)	8861 (2027)	8719 (2174)
Posterior sum of hair width	**Day 0**	**Day 180**	**Day 0**	**Day 180**
Mean (SD)	11894.4 (2838)	12 744 (2698)^a^, ^b^	12710.1 (2698)	12312.8 (2671)
Total sum of hair width	**Day 0**	**Day 180**	**Day 0**	**Day 180**
Mean (SD)	20867.1 (3474)	22152.1 (3709)^b^, ^c^	21571.2 (4120)	21032.8 (4405)
**Follicular units**	**Active treatment**		**Placebo**	
Anterior follicular units	**Day 0**	**Day 180**	**Day 0**	**Day 180**
Mean (SD)	114.2 (22.0)	117.5 (22.4)	115.8 (32.6)	111.1 (24.7)
Posterior follicular units	**Day 0**	**Day 180**	**Day 0**	**Day 180**
Mean (SD)	113.1 (26.4)	117.2 (28.3)	118.1 (25.7)	115.5 (27.1)
Total follicular units	**Day 0**	**Day 180**	**Day 0**	**Day 180**
Mean (SD)	227.4 (40.4)	234.7 (37.2)^d^	233.9 (54.9)	226.7 (47.3)

^a^ 2‐tailed Student's *t*‐tests for correlated samples *p* < 0.001.

^b^ *p* < 0.05 versus baseline.

^c^ 2‐tailed Student's *t*‐tests for correlated samples *p* < 0.01.

^d^ 2‐tailed Student's *t*‐tests for correlated samples *p* < 0.05.

For the total sum of follicular units per cm^2^, the mean change from baseline increased by 7.3 units/cm^2^ in the active treatment group and decreased by 7.2 units/cm^2^ in the placebo group by Day 180; the between‐group difference in mean change being significant (*p* < 0.05), representing a 201% (3‐fold) greater increase in follicular unit density with active treatment relative to placebo. This overall effect was supported by numerically favorable changes in both regions: in the anterior area, the mean change was +3.3 units/cm^2^ with active vs. –4.7 units/cm^2^ with placebo, and in the posterior area, +4.0 units/cm^2^ with active vs. –2.6 units/cm^2^ with placebo (Table 5).

#### 3.4.1 Secondary Endpoints, Male Subjects

In the male subgroup, the increase in the total sum of hair width was statistically significant and corresponded to an approximately 144% (2‐fold) greater improvement with active treatment vs. placebo. This overall effect was driven by the posterior region, where the mean change was +455.7 μm with active compared with −1137.5 μm with placebo by Day 180 (*p* < 0.01). In the anterior region, the mean change was +403.3 μm with active vs. –811.3 μm with placebo (Table 6).

**TABLE 6:** Secondary endpoint, sum of hair width and number of follicular units/cm^2^ for male treatment subgroup.

Sum of hair width	Active treatment, *n* = 40		Placebo, *n* = 20	
Anterior sum of hair width	**Day 0**	**Day 180**	**Day 0**	**Day 180**
Mean (SD)	9703.9 (1985)	10107.3 (1953)	9322.0 (2133)	8510.7 (2325)
Posterior sum of hair width	**Day 0**	**Day 180**	**Day 0**	**Day 180**
Mean (SD)	10484.0 (2617)	10939.6 (3058)^a^	12746.6 (2981)	11608.1 (2813)
Total sum of hair width	**Day 0**	**Day 180**	**Day 0**	**Day 180**
Mean (SD)	20187.9 (3695)	21046.9 (3970)^b^	22068.6 (4360)	20118.8 (4759)
Follicular units	**Active Treatment**		**Placebo**	
Anterior follicular units	**Day 0**	**Day 180**	**Day 0**	**Day 180**
Mean (SD)	112.5 (19.7)	117.6 (21.5)	117.4 (43.3)	110.7 (29.1)
Posterior follicular units	**Day 0**	**Day 180**	**Day 0**	**Day 180**
Mean (SD)	99.1 (18.8)	104.9 (25.6)	124.9 (29.5)	112.1 (31.5)
Total follicular units	**Day 0**	**Day 180**	**Day 0**	**Day 180**
Mean (SD)	211.7 (33.9)	222.5 (37.0)^c^	242.3 (69.3)	222.8 (58.7)

^a^ 2‐tailed Student's *t*‐tests for correlated samples *p* < 0.01.

^b^ 2‐tailed Student's *t*‐tests for correlated samples *p* < 0.05.

^c^ 2‐tailed Student's *t*‐tests for correlated samples *p* < 0.005.

For total follicular units in the male subgroup (Table 6), the mean change from baseline to Day 180 was +10.85 units/cm^2^ in the active treatment group versus −19.5 units/cm^2^ in the placebo group; the between‐group difference in mean change was statistically significant (*p* < 0.005), representing a 155% (2‐fold) greater increase in follicular unit density with active treatment relative to placebo. This overall effect was driven by the posterior region, which is a more problematic area for men; the mean change in posterior scalp was +5.8 units/cm^2^ with active compared with −12.8 units/cm^2^ with placebo (*p* < 0.05).

#### 3.4.2 Secondary Endpoints, Menopausal Female Subjects

In the menopausal female subgroup, the mean change in the total sum of hair width from baseline to Day 180 was +1383.6 μm in the active treatment group vs. –327.4 μm in the placebo group; the between‐group difference in mean change was significant (*p* < 0.05). This corresponded to a 606% (7‐fold) greater improvement in total hair width with active treatment compared with placebo. The overall effect was numerically supported by regional contributions: in the posterior area, the mean change was +1112.0 μm with active vs. +89.2 μm with placebo, and in the anterior area, which is a more problematic area in women, the mean change was +271.6 μm with active vs. –416.6 μm with placebo (Table 7).

**TABLE 7:** Secondary endpoint, sum of hair width for treatment menopausal female subgroup.

Sum of hair width	Active treatment, *n* = 40		Placebo, *n* = 20	
Anterior sum of hair width	**Day 0**	**Day 180**	**Day 0**	**Day 180**
Mean (SD)	8478.8 (1443)	8750.4 (1834)	7970.4 (1461)	7553.8 (1629)
Posterior sum of hair width	**Day 0**	**Day 180**	**Day 0**	**Day 180**
Mean (SD)	13494.6 (2332)	14606.6 (2336)	12692.8 (3339)	12782.0 (3439)
Total sum of hair width	**Day 0**	**Day 180**	**Day 0**	**Day 180**
Mean (SD)	21973.4 (3006)	23357.0 (3279)^a^	20663.2 (4755)	20335.8 (4875.5)

^a^ 2‐tailed Student's *t*‐tests for correlated samples *p* < 0.05.

#### 3.4.3 Investigator Hair Quality Global Improvement Scale Scores

At the Day 180 visit, the Investigator assessed improvements in subject hair quality compared to Day 0 using global photographs and clinical judgment based on hair quality parameters including hair brittleness, dryness, texture, shine, scalp coverage, and overall appearance using a 7‐point Likert scale. These data were analyzed when categorized as Improved (+1, +2, and +3), No Change (0), or Worsened (−1, −2, and −3) (Table 8). The Investigator rated the hair quality of 80% of subjects as Improved at Day 180 compared to Day 0.

**TABLE 8:** Numerical rating scale for self‐assessment questionnaire.

Rating	Description
7	Greatly Increased/Improved
6	Moderately Increased/Improved
5	Slightly Increased/Improved
4	No change
3	Slightly Decreased/Worsened
2	Moderately Decreased/Worsened
1	Greatly Decreased/Worsened

#### 3.4.4 Investigator Hair Growth Global Improvement Scale Scores

At the Day 180 visit, the Investigator also assessed changes in improvement in subject global hair growth as assessed relative to Baseline (Day 0) using global photographs and clinical judgment during the in‐person visit according to the same 7‐point Likert scale (Table 9). These data were also analyzed when categorized as Improved (+1, +2, and +3), No Change (0), or Worsened (−1, −2, and −3). Investigators rated 70% of subjects in the active treatment group as having increased hair growth at Day 180 compared to Day 0.

**TABLE 9:** Investigator ratings of change, day 180.

	Active(*n* = 40)	Placebo(*n* = 20)
**Hair quality, *n* (%)** ^a^		
Greatly improved	1 (2.5)	—
Moderately improved	12 (30)	—
Slightly improved	19 (47.5)	6 (30)
No change	4 (10)	9 (45)
Slightly worsened	4 (10)	4 (20)
Moderately worsened		1 (5)
Greatly worsened		
**Hair growth, *n* (%)** ^b^		
Greatly increased		
Moderately increased	8 (20)	1 (5)
Slightly increased	20 (50)	4 (20)
No change	6 (15)	7 (35)
Slightly decreased	6 (15)	6 (30)
Moderately decreased		2 (10)
Greatly decreased		

^a^ A two‐tailed binomial test of two proportions found differences in investigator improved hair quality ratings between supplement groups at Day 180 to favor the active group at each assessment, *p* < 0.0005.

^b^ A two‐tailed binomial test of two proportions found differences in investigator improved hair quality ratings between supplement groups at Day 180 to favor the active group at each assessment, *p* < 0.001.

#### 3.4.5 Hair Self‐Assessment Questionnaire

At Day 180, subjects were surveyed about 22 hair characteristics and asked to select the numerical rating that best described their perception of that characteristic compared to Day 0. Two skin characteristics were also assessed. Table 10 summarizes the positive ratings for each of the 22 hair and two skin characteristics at Day 180.

**TABLE 10:** Subjects with positive ratings^a^ for changes in hair and skin characteristics.

Hair/skin characteristic, *n* (%)	Active (*n* = 40)			Placebo (*n* = 20)		
	Day 56	Day 90	Day 180	Day 56	Day 90	Day 180
Hair shedding	17 (42.5)	16 (40)	19 (47.5)	11 (55)	13 (65)	14 (70)
Overall hair growth	20 (50)	28 (70)	28 (70)	9 (45)	10 (50)	13 (65)
Overall hair volume	13 (32.5)	22 (55)	27 (67.5)	5 (25)	10 (50)	12 (60)
Scalp coverage	15 (37.5)	21 (52.5)	24 (60)	4 (20)	9 (45)	9 (45)
Hair thickness	15 (37.5)	17 (42.5)	25 (62.5)	5 (25)	7 (35)	7 (35)
Hair fullness	18 (45)	19 (47.5)	25 (62.5)	5 (25)	7 (35)	11 (55)
Hair quality	12 (30)	25 (62.5)	21 (52.5)	7 (35)	9 (45)	11 (55)
Hair shine/gloss	11 (27.5)	17 (42.5)	14 (35)	5 (25)	8 (40)	9 (45)
Hair strength	18 (45)	22 (55)	19 (47.5)	8 (40)	10 (50)	11 (55)
Hair softness	16 (40)	22 (55)	14 (35)	6 (30)	6 (30)	10 (50)
Amount of noticeable new hair	14 (35)	22 (55)	25 (62.5)	4 (20)	10 (50)	12 (60)
Hair growth rate	17 (42.5)	22 (55)	23 (57.5)	4 (2)	10 (50)	13 (65)
Hair growth on top of head	17 (42.5)	21 (52.5)	26 (65)	6 (30)	10 (50)	8 (40)
Hair growth of hairline	13 (32.5)	13 (32.5)	19 (47.5)	4 (20)	8 (40)	11 (55)
Hair length	14 (35)	20 (50)	23 (57.5)	5 (25)	7 (35)	9 (45)
Ease of styling	11 (27.5)	13 (32.5)	14 (35)	5 (25)	5 (25)	5 (25)
Hair Moisture	12 (30)	15 (37.5)	13 (32.5)	9 (45)	5 (25)	5 (25)
Overall hair appearance	18 (45)	22 (55)	24 (60)	12 (60)	11 (55)	12 (60)
Growth of eyebrow hair	8 (20)	14 (35)	14 (35)	20 (100)	4 (20)	4 (20)
Thickness of eyebrow hair	7 (17.5)	13 (32.5)	14 (35)	20 (100)	20 (100)	4 (20)
Growth of eyelash hair	2 (5)	6 (15)	5 (12.5)	1 (5)	1 (5)	3 (15)
Thickness of eyelashes	1 (2.5)	4 (10)	3 (7.5)	1 (5)	1 (5)	3 (15)
Skin smoothness and fine lines	4 (10)	6 (15)	4 (10)	6 (30)	4 (20)	6 (30)
Overall skin health	5 (12.5)	4 (10)	4 (10)	5 (25)	6 (30)	8 (40)

^a^ Positive denotes greatly, moderately, and slightly improved subject ratings of change.

Among subjects in the active treatment group, most ratings (66.7%) trended upward across Day 180 assessments. Overall positive ratings were numerically superior for the active treatment group at each assessment period. At Day 180, hair thickness was significantly greater for the active treatment group (62.5% versus 35% in placebo, *p* < 0.05).

#### 3.4.6 Hair Thinning Quality of Life Questionnaire

Subjects responded to the Hair Thinning Quality of Life Questionnaire on Days 0 and 180. Possible responses to each question were 4, Very Much; 3, A Lot; 2, A Little; 1, Not at All; or 0, Not Relevant.

1. I am embarrassed by my thinning hair.
2. My thinning hair impacts my self‐esteem.
3. My thinning hair makes me feel self‐conscious.
4. My thinning hair makes me less outgoing than I would like to be.
5. My thinning hair makes me feel unattractive.
6. My thinning hair makes me feel stressed.
7. My thinning hair makes me feel anxious.

Among subjects in the active treatment group, a trend analysis showed a steady improvement across successive assessments for *My thinning hair makes me feel unattractive* and *My thinning hair makes me feel anxious* (*p* < 0.05). There were no significant changes for subjects in the placebo group. When the active treatment group was compared with the placebo group, there was a significantly greater number of active treatment subjects that showed improvement on the statement *My thinning hair makes me feel stressed* (*p* < 0.05).

#### 3.4.7 Safety Endpoint, Day 180

There were no reported local or systemic adverse events considered probably or possibly related to the investigational product.

## 4 Discussion

To meet the needs of subjects seeking treatment for male and female pattern hair loss, a novel proprietary extract of bioactive fatty acids from *S. repens* (saw palmetto) has been formulated for treating hair loss and maintaining hair health [7]. In a recently published research article, SEREVELLE (formerly USPlus DERM) was found to support hair growth via a dual pathway. First, its enriched free fatty acid complex potently inhibits 5‐α‐reductase‐I in human dermal papilla cells (IC₅₀ < 0.39 μg/mL) and found to be 75X more potent in reducing the 5‐α‐reductase‐I than standard lipidosterolic extract of saw palmetto. Second, SEREVELLE also prolongs the anagen hair cycle phase as observed in ex vivo human follicles cultured without testosterone, reduces melanin clumping (a marker of organ‐culture stress/oxidative load), trends toward higher hair‐matrix keratinocyte activity, and reinforces the epithelial stem‐cell niche (increased K15 expression) with maintained quiescence. SEREVELLE's distinct lipid composition (ActiLipid4Hair) and its follicular effects promote a healthier, longer growth phase through both 5α‐reductase dependent and independent mechanisms [12].

Prior to the present study, topical application of this bioactive fatty acid extract was assessed in a 12‐weeek duration in subjects with ongoing hair loss for ≥ 6 months [7]. At Week 12, most subjects (90.9%, *p* = 0.0001) noted improvements in hair density (22.4%, *p* = 0.067) and general hair loss (19.5%, *p* = 0.001). Female subjects achieved a significant 25% decrease in hair loss (*p* = 0.004). Subject satisfaction was high.

Subsequently, a 6‐month randomized, double‐blind, placebo‐controlled study of the oral bioactive extract was launched [8]. The objective of this 180‐day assessment to was to investigate the long‐term efficacy of the bioactive fatty acid saw palmetto extract to promote hair growth in adult men and women with self‐perceived hair thinning.

The results showed that subjects maintained anterior, posterior, and total terminal hair growth at Day 180. Results at 180 days showed significant 4‐fold improvement in total terminal hair count (283%, *p* < 0.001), 5‐fold increase in total vellus hair count (414%, *p* < 0.05), and 4‐fold increase in total hair density (306%, *p* < 0.001). There was also a significant 5‐fold increase for total hair width (thickness) (*p* < 0.01) and 3‐fold increase in the number of follicular units (*p* < 0.05). These results indicate follicle activation and new hair growth, thicker mature hairs, greater hair density and coverage, and more visible hair fullness. Importantly, the advantage of active treatment over placebo continued to widen after 90 days till the end of the trial. When the net treatment effect vs. placebo was compared between two time points (90 days and 180 days), the treatment benefit at Day 180 was approximately ~40% larger than the benefit observed on Day 90.

Among menopausal female subjects in the active treatment group, sustained growth was maintained throughout the 180‐day study duration. Compared to placebo, there was a 4‐fold (312%) increase in terminal hairs, a 2‐fold increase in vellus hairs (117%), and an overall 3‐fold growth in total hairs (230%), or total hair density. There was also a 3‐fold (209%) increase in the number of follicular units compared to placebo treatment, and a 7‐fold (668%) increase in total hair width (thickness). These results indicate increased hair follicles, follicle activation, and new hair growth, stronger and thicker mature hairs, and greater hair density scalp coverage for female patients.

Among male subjects in the active treatment group, sustained growth throughout the 180‐day study duration was observed. Significant improvements include a 2‐fold increase in total terminal hairs (154%, *p* < 0.01), a nearly 4.7‐fold increase in total vellus hairs (378%, *p* = 0.047), almost a 3‐fold increase in total hair density (192%, *p* < 0.001), a 2‐fold (144%) increase in the total sum of hair width (thickness, *p* < 0.05), and a 2.5‐fold (155%) increase in the number of follicular units (*p* < 0.01).

It is noteworthy that these results suggest the beneficial effects of the bioactive fatty acid extract from *S. repens* are durable and may provide ongoing clinical improvements with continued use.

In contrast to a recent shorter‐duration study of topical and oral saw palmetto oil [13] that focused primarily on hair loss, our 180‐day, placebo‐controlled trial employed high‐resolution trichoscopy and comprehensive clinical and patient‐reported measures, demonstrating robust, statistically significant improvements in hair density, thickness, and hair‐related quality of life [8]. Previously, a review of five randomized clinical trials and two prospective cohort studies also provided evidence of positive effects following topical and oral treatment with saw palmetto‐containing supplements among subjects with androgenetic alopecia and telogen effluvium [5]. Hair improvements included overall hair quality, total hair count, and hair density.

To our knowledge, this is the first clinical study with a saw palmetto extract to employ an intensive trichoscopic protocol quantifying both terminal and vellus hairs in anterior and posterior scalp regions in men and women. Previous studies have only reported terminal and vellus hair counts in more limited scalp areas. The study also proves that this proprietary extract of saw palmetto containing bioactive free fatty acids (ActiLipid4Hair) is safe as a health supplement for use in both men and women for a longer duration.

In the present clinical study, the proprietary saw palmetto extract containing bioactive free fatty acids (ActiLipid4Hair complex) produced significant improvements in terminal hair growth parameters. Complementary ex vivo works with the same formulation demonstrated an approximately 30% increase in the proportion of hair follicles in anagen phase, supporting a mechanistic role in promoting a healthier hair cycle. This may have relevance for individuals experiencing or at risk for telogen effluvium, including those treated with GLP‐1‐based therapies, where rapid weight loss and metabolic changes have been associated with increased shedding. Although GLP‐1 users were not specifically evaluated in this trial, the combination of terminal hair gains *in* vivo and anagen prolongation ex vivo provides a biologically plausible rationale for future studies in this population.

This study further reiterates mechanistic advantages and clinical benefits over other hair growth ingredients like biotin, which primarily supports the growth and quality of existing hair. Saw palmetto extract, such as one used in this clinical study, containing a bioactive free fatty acids complex of ActiLipid4Hair, shows thickening of increase in hair count and thickness of existing hair as well as emergence of new hair (vellus hair).

There were no reports of local or systemic adverse events considered to be possibly or probably related to the bioactive fatty acids extract at any time during the 6‐month study. Two reports of headaches occurred at the final Day 180 assessment; however, they were transient and unrelated to study treatment. These findings are also in agreement with previous work [5, 8].

## 5 Conclusion

The results of this 6‐month randomized double‐blind, placebo‐controlled study provide evidence that the daily oral administration of a proprietary formulation of a bioactive fatty acid extract from saw palmetto ( *Serenoa repens* ) provides beneficial effects for men and women with self‐perceived hair loss. The primary endpoint analysis revealed statistically significant and clinically meaningful improvements in anterior and posterior terminal, vellus, and total hair counts at study Day 180 for the active treatment group vs. placebo, strongly supporting the positive long‐term treatment effect on hair growth in men and women. In addition, improvements observed at Day 90 were maintained through Day 180, and the difference between active treatment and placebo continued to widen over this period, indicating sustained benefit for longer‐term use.

## Conflicts of Interest

The author declares no conflicts of interest.

## Data Availability

The data that support the findings of this study are available from the corresponding author upon reasonable request.
